# Agricultural biotechnology in China: product development, commercialization, and perspectives

**DOI:** 10.1007/s42994-025-00209-4

**Published:** 2025-05-15

**Authors:** Jingang Liang, Yu Sun, Yanchao Yang, Zeyu Wang, Han Wu, Taotao Gu, Ruifu Zhang, Xinli Sun, Bin Yao, Tao Tu, Xiaoqing Liu, Huiying Luo, Guangzhi Tong, Yue Jiao, Kui Li, Jie Zhang, Kongming Wu

**Affiliations:** 1 Development Center of Science and Technology, Ministry of Agriculture and Rural Affairs, Beijing, 100176 China; 2 Biotechnology Research Institute, Shanghai Academy of Agricultural Sciences, Shanghai, 201106 China; 3 State Key Laboratory for Biology of Plant Diseases and Insect Pests, Institute of Plant Protection, Chinese Academy of Agricultural Sciences, Beijing, 100193 China; 4 Shenzhen Branch, Guangdong Laboratory for Lingnan Modern Agriculture, Key Laboratory of Synthetic Biology, Ministry of Agriculture and Rural Affairs, Agricultural Genomics Institute at Shenzhen, Chinese Academy of Agricultural Sciences, Shenzhen, 518120 China; 5 School of Life Sciences, Henan University, Kaifeng, 475004 China; 6 Shenzhen Research Institute of Henan University, Shenzhen, 518000 China; 7 College of Resources and Environmental Sciences, Nanjing Agricultural University, Nanjing, 210095 China; 8 Institute of Agricultural Resources and Regional Planning, Chinese Academy of Agricultural Sciences, Beijing, 100081 China; 9 Institute of Animal Sciences of Chinese Academy of Agricultural Sciences, Beijing, 100193 China; 10 Shanghai Veterinary Research Institute, Chinese Academy of Agricultural Sciences, Shanghai, 200241 China

**Keywords:** Agricultural biotechnology, China, Genetically modified organisms, Product development, Commercialization, Perspectives

## Abstract

Meeting the increasing demand for food and industrial products by the growing global population requires targeted efforts to improve crops, livestock, and microorganisms. Modern biotechnology, particularly genetic modification (GM) and genome-editing (GE) technologies, is crucial for food security and environmental sustainability. China, which is at the forefront of global biotechnological innovation and the rapid advancements in GM and GE technologies, has prioritized this field by implementing strategic programs such as the National High-tech Research & Development Program in 1986, the National Genetically Modified Organism New Variety Breeding Program in 2008, and the Biological Breeding-National Science and Technology Major Project in 2022. Many biotechnological products have been widely commercialized in China, including biofertilizers, animal feed, animal vaccines, pesticides, and GM crops such as cotton (*Gossypium hirsutum*), maize (*Zea mays*), and soybean (*Glycine max*). In this review, we summarize progress on the research and utilization of GM and GE organisms in China over the past 3 decades and provide perspectives on their further development. This review thus aims to promote worldwide academic exchange and contribute to the further development and commercial success of agricultural biotechnology.

## Introduction

Biotechnology has become indispensable in agriculture, providing substantial economic, social, and ecological benefits. Since the first commercial cultivation of genetically modified (GM) crops in the United States in 1996, the global application of biotechnology and genetically modified organisms (GMOs) has grown substantially. This development has established biotechnology, particularly transgenic technology, as a pivotal force propelling a new scientific and technological revolution in agriculture and as a critical strategy for scientific innovation (ISAAA 2019). Global cultivation of GM crops has demonstrated their advantages for economic development and environmental sustainability (Raman 2017). For example, cultivating insect-resistant and herbicide-tolerant GM crops significantly diminishes pesticide usage during the crop growth period while effectively managing pests and improving crop yields (Dong et al. 2021; Kuang et al. 2024; Zhang et al. 2016). Furthermore, biotechnology has advanced the development of animal breeding materials, vaccines, feed additives, and other solutions aimed at enhancing animal husbandry efficiency while reducing environmental impacts (Shakweer 2015; Shakweer et al. 2023). The use of transgenic, genome editing (GE), whole-genome selection, synthetic biology, and other technologies to conduct efficient, accurate, and targeted improvement and breed animals, plants, and microorganisms is key to agricultural innovation (Wang 2022). For example, microbial biofertilizers represent a promising solution for sustainable agriculture, offering benefits such as enhanced soil fertility, improved crop yields, and lower environmental impacts. Agricultural biotechnology is also important for achieving a high level of scientific and technological self-reliance in developing new varieties, and promoting increased and stable agricultural output to ensure food security. The extent of research and implementation of these technologies is an important indicator of the agricultural competitiveness of a given country.

China has made remarkable strides in agricultural biotechnology, particularly in bio-breeding, i.e., the application of biotechnology to enhance the genetic traits of animals, plants, and microorganisms. Many nationally funded projects have been established to date (Li et al. 2014). For example, the National High-tech Research & Development Program (namely, the 863 Program) and the National Key Basic Research Program (namely, the 973 Program) were initiated in 1986 and 1997, respectively; the National Genetically Modified Organism New Variety Breeding Program, representing the major scientific and technological project in the agricultural field, was launched in 2008; and the Biological Breeding-National Science and Technology Major Project was established in 2022. Moreover, bio-breeding was elevated to a national strategic priority within the 14th Five-Year Plan and Vision 2035, transitioning from “orderly promotion” to “expanded scope and accelerated pace,” as outlined in Central Document No. 1 (Zhang et al. 2022b). In 2022, to systematically promote the industrial application of bio-breeding, modernize the seed industry, and ensure food security, the Ministry of Agriculture and Rural Affairs of the People’s Republic of China (MARA) revised four regulations on the management of agricultural GMOs (MARA 2022c), issued the “Measures for Reviewing and Approving Genetically Modified Maize and Soybean Varieties,” and granted approval biosafety certificates for these varieties (MARA 2022d, 2023a, 2024c).

In this review, we examine the current status of research and applications of agricultural GMOs in China, analyze future prospects and challenges, and discuss strategies to promote the development and utilization of agricultural biotechnology in China.

## Current progress in agricultural biotechnology research

### Discovery of important functional genes

The genetic modification of agricultural organisms relies on adding, removing, and/or modifying the genes and genomic regions underlying key traits. Advancements in molecular biology and genomic techniques have helped researchers explore topics ranging from understanding gene functions to applied innovations in bio-breeding programs. Gene cloning has progressed from small-scale efforts to large-scale discovery of genes with significant value for breeding programs. In this section, we describe three types of genes that have been extensively studied for use in GMOs: biotic stress-resistance genes, genes that improve nutrient quantity and quality, and abiotic stress-resistance genes.

#### Genes that improve biotic stress resistance

The presence of insect pests, weeds, and diseases significantly affects crop yields and stability. Effective prevention and control measures are essential for ensuring optimal yield per unit area as well as total production. The classic insecticidal crystalline (Cry) proteins from *Bacillus thuringiensis* (*Bt*) confer resistance to specific orders of insects (generally Lepidoptera). In recent years, Chinese scientists have explored new *cry* genes, including *cry9Cb1* (Shan et al. 2019), *cry1A*, and *cry2A*, along with the *B. thuringiensis* gene *vip3A*, which encodes an insecticidal protein unrelated to Cry proteins; these genes are widely used to control Lepidopteran insects. In addition, *cry3B1* and *cry34/35Ab1* are used to target Hemipteran insects, and *cry51Aa2* is used to control Dipteran insects. In addition, other innovative plant-derived insecticidal proteins have been discovered, such as those sourced from ferns (Wei et al. 2023) and the cotton (*Gossypium hirsutum*)-derived jasmonate ZIM-domain (JAZ) protein GhJAZ24 (Mo et al. 2024). These proteins are structurally similar to known Cry proteins from *B*. *thuringiensis* yet possess unique characteristics and have been shown to be effective against Lepidopteran pests, thereby offering expanded strategies for pest control.

Weed control remains a major challenge in agricultural production. The incorporation of herbicide tolerance (HT) genes into crops has provided efficient solutions for simplified cultivation practices and limits soil and fertilizer loss. However, the evolution of herbicide-resistant weeds remains a challenge for HT traits. Resistance to glyphosate, the most common HT trait, is conferred by genes from different organisms that encode the enzyme 5-enolpyruvylshikimate-3-phosphate synthase (EPSPS), which is targeted by glyphosate (Li et al. 2020b; Sauer et al. 2016). EPSPS genes such as *cp4 epsps* (from *Agrobacterium* sp. strain CP4) and *G2-epsps* (from *Pseudomonas fluorescens* strain G2), in addition to genes that encode proteins that can modify and thus detoxify glyphosate, such as glyphosate oxidoreductase (*gox*) and acetyltransferase (*gat*) genes, enable glyphosate resistance (Li 2023; Wang et al. 2023). Several additional HT genes have been developed and integrated into commercially available GM crops in China, including EPSPS-like genes (*2m-epsps*, *g10evo-epsps*, *gr79-epsps*, *am79-epsps*) and cytochrome P450 gene from Bermuda grass (*Cynodon dactylon*; *CdP450*). For other herbicides, mutations conferring HT have been identified in genes that encode herbicide targets such as acetolactate synthase (Bottero et al. 2022; Kuang et al. 2020; Li et al. 2016; Perroud et al. 2021; Ran et al. 2018; Wu et al. 2020a; Xu et al. 2021b; Yan et al. 2021), acetyl-CoA carboxylase (Li et al. 2020a; Xu et al. 2020b, 2021b, c; Yan et al. 2021), protoporphyrinogen oxidase, and 4-hydroxyphenylpyruvate dioxygenase (Lu et al. 2021; Wu et al. 2023).

GE, which allows precise and efficient modification of genomic loci to create elite crop varieties with desired traits, provides another strategy for developing HT (Dong et al. 2021). Researchers have utilized multiple approaches such as saturated targeted endogenous mutagenesis editors (Li et al. 2020a), base-editing-mediated gene evolution (Kuang et al. 2020), and plant dual-base editor version 1 (Xu et al. 2021b) to induce near-saturated mutations in target loci, successfully identifying new mutations associated with HT. These advancements in GE enable highly efficient editing for complex genetic enhancements, while reducing off-target effects, and faster screening for mutation types that better match expected target traits, underscoring its transformative potential for enhancing agricultural sustainability and weed management, providing an effective strategy for developing crops with tailored HT profiles.

Plant diseases caused by viruses, bacteria, fungi, and nematodes are another major challenge in agricultural production. Using plant-derived disease resistance genes to develop disease-resistant varieties presents a viable alternative to conventional measures such as the use of pesticides. In recent years, several important disease resistance genes have been identified, including the broad-spectrum rice blast resistance gene *Pigm* in rice (*Oryza sativa*) (Deng et al. 2017); the recessive resistance gene *Rab GDP dissociation inhibitor alpha* (*ZmGDIα*) against maize (*Zea mays*) rough dwarf disease (Liu et al. 2020a; Zhong et al. 2024); the major-effect locus *Resistance to Fusarium graminearum 1* (*qRfg1*, harboring a transposon insertion in the CCT domain-containing gene *ZmCCT*) conferring maize stalk rot resistance (Wang et al. 2017); the quantitative trait locus *Multiple drug resistance 9.02* (*qMdr9.02*) in *caffeoyl*‐*CoA O‐methyltransferase 2* (*ZmCCoAOMT2*), which provides dual resistance against corn small spot and gray spot diseases (Yang et al. 2017); the maize gene *Wall-associated kinase* (*ZmWAK*) against silk black rust (Zuo et al. 2015); the broad-spectrum powdery mildew resistance genes *Pm13*, *Pm36*, and *Pm57* in wheat (*Triticum aestivum*) (He et al. 2024; Li et al. 2024b; Zhao et al. 2024); and the broad-spectrum Asian soybean rust resistance gene pair *Rpp6907-7* and *Rpp6907-4* (Hao et al. 2024). With the rapid advancement of GE and whole-genome selection-assisted breeding technology, these genes are expected to find broader applications, contributing to the improvement of disease resistance in crops and the development of superior crop varieties.

In addition to the successes of GM plants, GE has effectively been used in animal agriculture. Porcine reproductive and respiratory syndrome virus (PRRSV) causes severe economic losses to the global swine market, making genetic improvement for resistance against this virus particularly crucial. As an example of generating resistance by editing a disease susceptibility locus, using clustered regularly interspaced short palindromic repeats (CRISPR)/CRISPR-associated nuclease 9 (Cas9) technology, Yang et al. (2018) created pigs with knockout mutations in the putative PRRSV receptor gene *CD163*; these animals are unable to express CD163 protein on the surface of macrophages, thus blocking PRRSV from invading porcine respiratory cells, producing complete resistance to highly pathogenic PRRSV infection. Subsequently, the researchers performed multi-gene editing to obtain pigs resistant to multiple viruses, the gene editing of *CD163* and *porcine aminopeptidase N* (*pAPN*) in pigs resulted in simultaneous resistance to infections by PRRSV, porcine-transmissible gastroenteritis virus, and porcine delta-coronavirus (Xu et al. 2020a). Additional studies have focused on the genetic enhancement of disease resistance across various animal species, including cows engineered to express a human lysozyme gene to combat mastitis and cows with edited versions of the *SP110* (*Speckled 110kDa*, also known as *intracellular pathogen resistance 1*, *Ipr1*) gene to confer resistance to tuberculosis (Wu et al. 2015). To bolster disease resistance, China Agricultural University developed transgenic goats overexpressing *Toll-like receptor 2* (*TLR2*) and transgenic sheep overexpressing *Toll-like receptor 4* (*TLR4*). In these animals, TLRs induce autophagy pathways, enhancing the removal of *Staphylococcus aureus* (Deng et al. 2012, 2013; Wang et al. 2022).

Classic examples of disease resistance in aquaculture include rainbow trout (*Oncorhynchus mykiss*) carrying the gene *cecropin* B (*CB*), which produced specific resistance to pathogens such as *Aeromonas salmonicida*, infectious *hematopoietic necrosis virus*, and *Vibrio anguillarum* (Chiou et al. 2014; Han et al. 2018). Zebrafish (*Danio rerio*) carrying the genes *chelonianin* and *tilapia hepcidin 1–5* (*TH1-5*) showed significantly increased survival rates following infection with *Vibrio vulnificus* and *Streptococcus agalactiae* (Pan et al. 2011). Turbot (*Scophthalmus maximus*) carrying *Paralichthys olivaceus* C-type lysozyme gene demonstrated antibacterial activity (Ji and Zhang 2004). Grass carp (*Ctenopharyngodon idella*) with *interferon* (*IFN*) or *lactoferrin* genes exhibited enhanced resistance to grass carp reovirus (Mao et al. 2004). Rare minnows (*Gobiocypris rarus*) carrying the *myxovirus resistance* (*Mx*) gene showed improved resistance against hemorrhagic disease (Su et al. 2009).

#### Genes that improve nutritional quantity and quality

One major challenge in crop production is improving nitrogen use efficiency to decrease the need for costly inputs and limit fertilizer runoff, a major source of pollution. A research consortium identified the high-yield-related gene *Dehydration-Responsive Element-Binding Protein 1C* (*OsDREB1C*), which enhances both photosynthetic efficiency and nitrogen utilization in rice (Wei et al. 2022). The discovery of the gene *nitrogen-mediated tiller growth response 5* (*NGR5*) in rice paved the way for innovative breeding strategies aimed at enhancing grain output while simultaneously optimizing nitrogen use efficiency. This approach minimizes the reliance on synthetic fertilizers and mitigates environmental pollution, contributing to the development of innovative, eco-friendly, high-yielding, and efficient crop varieties (Wu et al. 2020b).

Another major challenge is improving plant architecture to allow efficient light capture in high-density plantings. The plant architecture of maize is governed by the gene *leaf angle architecture of smart canopy 1* (*lac1*), which fosters a “top tight-bottom loose” configuration, and the gene *RAV1-like 1* (*RAVL1*), which encourages a “compact top and bottom” structure; these genes hold significant promise for high-density planting strategies and yield enhancement (Tian et al. 2024). In rice, high-plant-height heterosis in *indica* × *japonica* hybrid is a common problem, and DWARF53 (D53) functions as an inhibitor within the strigolactone signaling pathway that regulates tiller growth and development, thereby decreasing plant height. This finding broadens the prospects for the effective utilization of heterosis in *indica* × *japonica* hybrid rice to enhance agricultural production (Jiang et al. 2013).

Enhancing the production performance of agricultural animals, for factors such as meat yield, growth rate, feed efficiency, and reproduction, has been a primary breeding objective for livestock. The gene *myostatin* (*MSTN*) induces the double-muscled phenotype in cattle, which significantly enhances muscle mass and meat yield (Grobet et al. 1997). This finding holds promise for addressing the growing global demand for animal protein while optimizing the utilization of feed resources. *MSTN* has been successfully used to increase the yield of cattle and the fish species Nile tilapia (*Oreochromis niloticus*) and red sea bream (*Pagellus bogaraveo*). These animal products have undergone regulatory review in Argentina, Brazil, and Japan (Ledesma and Van Eenennaam 2024). China has initiated the development of *MSTN*-edited pigs through GE to bolster meat production. Fan et al. (2022a) explored how different target sites in *MSTN* can be employed to counteract hind limb weakness and explore changes in feed conversion, reproduction, and meat quality in *MSTN-*edited pigs across distinct genetic backgrounds (Fan et al. 2022a). Similar work targeting *MSTN* has been performed in ruminants and rodents (Crispo et al. 2015; Ding et al. 2020; Han et al. 2014; Luo et al. 2014; Proudfoot et al. 2015; Yu et al. 2016; Zhang et al. 2018b, 2019a).

Another focal point of current research lies in increasing the contents of polyunsaturated fatty acids (PUFAs) in livestock meat and freshwater fish. Omega-3 PUFAs (n-3 PUFAs), which play a pivotal role in human health, are primarily obtained from marine fish because most vertebrates lack the capacity to biosynthesize n-3 PUFAs and so must rely on dietary intake. Introducing a pig codon-optimized *fatty acid desaturase-1* (*fat-1*) from a nematode (*Caenorhabditis elegans*) into pigs substantially boosted n-3 PUFA levels in pork (Tang et al. 2014). In pigs with the *fat-1* gene, the level of n-3 PUFAs was about 3.1-fold higher in the muscle tissues compared with those in the wild type (Ren et al. 2011). In another study, genes encoding omega-3 fatty acid desaturase and ∆12 fatty acid desaturase from the same nematode species were introduced into yellow river carp (*Cyprinus carpio haematopterus*). The transgenic carp were able to biosynthesize PUFAs and showed an evident decrease in n-6 PUFA contents and a substantial increase in n-3 PUFA contents, significantly improving the quality of this fish species (Zhang et al. 2019b).

#### Genes that improve abiotic stress resistance

Due to global climate change and secondary anthropogenic activities, crops and animals are facing increasing pressure from abiotic stresses such as high temperatures, drought, and saline-alkaline soils. Moreover, factors such as drought and high-temperature stress often occur simultaneously, affecting organism growth and productivity. Genetic engineering plays a significant role in mitigating sensitivity to environmental stress and enhancing abiotic stress resistance in both crops and animals. Over the past 3 decades, a substantial number of abiotic stress–specific genes and transcription factors have been discovered and characterized in diverse plant species, some of which exhibit a common regulatory mechanism in response to multiple stresses, while others are responsible for specific stress responses. Remarkable progress has been made in developing GMOs with enhanced abiotic stress resistance, thereby improving their adaptability to adverse environmental conditions.

For example, GM plants and animals have been engineered to better tolerate heat stress. Heat shock proteins are molecular chaperones that play key roles in response to abiotic stresses such as high/low temperature and drought (Saeed et al. 2019). Some genes specifically involved in heat stress have also been discovered, for example, the *thermo-tolerance 3* (*TT3*) locus, which was identified from African cultivated rice (CG14), comprises two genes: *TT3.1* and *TT3.2*. These two genes transduce heat signals from plasma membrane to chloroplasts, and antagonistically regulate heat stress resistance in rice, overexpressing *TT3.1* or knocking out *TT3.2* conferred significant yield increases under heat stress (Zhang et al. 2022a). The dehydration response element binding protein (DREB) is a transcription factor involved in abiotic stress responses (Zhang and Xia 2023). Homologous *DREB* genes have been discovered in various plant species, including soybean (*Glycine max*) (Chen et al. 2009), wheat (Mei et al. 2022), cotton (Li et al. 2017), and maize (Qin et al. 2004). Overexpression of these genes in GM plants has been shown to increase tolerance to drought, high salt, and cold stresses. In livestock, the *prolactin receptor* (*PRLR*) gene was edited to produce slick-haired cattle with enhanced heat dissipation, potentially improving animal welfare and production efficiency in warmer climates (Rodríguez-Villamil et al. 2021). *PRLR* was successfully used to produce heat-tolerant dairy cattle and beef cattle, and the cattle products have undergone regulatory review in Argentina, Brazil, and the United States (Ledesma and Van Eenennaam 2024).

Soil salinization causes serious damage to plant tissues. Plants have evolved flexible mechanisms to cope with salinity stress through morphological, physiological, biochemical, and molecular adaptations. Genes and transcription factors involved in ion homeostasis and compartmentalization, osmotic adaptation, and increased antioxidation metabolism have been intensively studied (Balasubramaniam et al. 2023). For instance, vacuolar Na^+^/H^+^ antiporter (NHX) proteins function as monovalent ion exchangers that sequester Na^+^ into the vacuole in exchange for H^+^. Since *AtNHX1* from *Arabidopsis thaliana* was discovered (Apse et al. 1999), it has been heterologously expressed in other plant species to increase their salt tolerance, such as in tomato (*Solanum lycopersicum*) (Zhang and Blumwald 2001), tobacco (*Nicotiana tabacum*) (Duan et al. 2009), cotton (Shen et al. 2015), wheat (Xue et al. 2004), soybean (Cao et al. 2011), and rice (Chen et al 2007). Other well-characterized genes involved in salt tolerance include salt overly sensitive genes, high-affinity potassium transporters (HKTs) genes, and proton pump genes. The *alkaline tolerance 1* (*AT1*) gene holds significant potential for improving salinity tolerance in various cereal crops and is expected to play an important role in supporting the comprehensive utilization of saline-alkali lands in China’s national food security strategy (Zhang et al. 2023a). Co-overexpression of these genes is a promising strategy to achieve higher salt tolerance than using a single gene (Balasubramaniam et al. 2022).

### Innovation in biotechnology techniques

Efficient, rapid, and precise breeding technologies are important for enhancing the competitiveness of the seed commercialization. Through the implementation of national strategic projects, China’s R&D capabilities now rank among the best worldwide, as several key technical bottlenecks have been overcome to form a scaled transgenic technology system. Significant breakthroughs have also been achieved in GE technologies, evolving from basic tools to precise editing techniques. The efficiency of genetic transformation in major animals and plants has reached an advanced level worldwide. Techniques such as haploid breeding, haploid induction (Li et al. 2021; Liu et al. 2017; Zhong et al. 2019), and artificial apomixis (Zhang et al. 2023b) lay the foundation for the precise design breeding and domestication of crops (Qu et al. 2024). New technologies such as fluorescence-labeled intelligent male sterility in major crops (Chang et al. 2016; Zhang et al. 2018a) bring many opportunities for innovations in germplasm development. Genomics and phenomics techniques make it possible to design and select new crop varieties at the whole-genome level. Furthermore, molecular breeding technologies greatly improve the effectiveness of breeding (Xu et al. 2014; Yu et al. 2023).

GE technologies have continuously advanced, achieving a leap from random alterations to precise editing and even large-scale DNA manipulation. The CRISPR/Cas system has become mainstream due to its simplicity and robust programmability. Base editors can now be used to convert bases, transforming targeted GE from random mutagenesis to precise single-base substitutions. Prime editors can be used for small fragment editing, and technologies such as dual prime editing guide RNA expanded the ability to insert or delete large DNA segments (Li et al. 2024a; Pacesa et al. 2024). Jinsheng Lai’s team at China Agricultural University discovered the nucleases Cas12i and Cas12j, which obtained patent authorizations from the National Intellectual Property Administration in April and May 2021, respectively (Chinese patents ZL201980014560.3 and ZL201980014005.0). The outstanding contributions of Professor Caixia Gao’s team in designing deaminase and non-CRISPR base editors are expected to help rid China of the shackles of CRISPR patents. Additional innovations are expected to improve the efficiency of GE tools in mammalian cells and embryos. For example, as the ancestor of Cas9, the insertion sequences Cas9-like OrfB (IscB) is an RNA-guided nuclease, which associates with ωRNA to be guided to the target loci and subsequently cleaves double-stranded DNA targets, introducing three substitutions into IscB led to an average 7.5-fold increase in activity in mammalian cells (Xue et al. 2024).

Biotechnology relies on the ability to deliver genes, including transgenes and reagents for CRISPR/Cas-mediated GE. The development of traditional Agrobacterium (*Agrobacterium tumefaciens*)*-*mediated plant transformation and bombardment with gene guns was followed by innovative methods such as virus-mediated delivery, tissue culture-free delivery, and delivery via grafting. The use of regeneration-associated genes such as *Baby boom* (*ZmBBM*), *Wuschel 2* (*ZmWUS2*) (Johnson et al. 2023), *GRF-interacting factor* (*GIF*) and *growth-regulating factor* (*GRF*) (Yarra and Krysan 2023), and *WUS-related homeobox 5* (*TaWOX5*) (Harwood 2023) has been explored to enhance the efficiency of plant genetic transformation and broaden the range of genotypes amenable for transformation, thereby addressing the bottleneck of genotype dependency. A series of studies have successfully delivered DNA and RNA macromolecules into plant cells using carbon nanomaterials, which do not exert external force on the plants or cause tissue damage. It is possible to produce stable genetically edited plants without the need for plant regeneration to form callus tissue, marking a significant advancement in plant genetic transformation (Demirer et al. 2019a, b; Kwak et al. 2019). These advancements have greatly improved plant transformation efficiency, expanding the range of species or varieties that can be improved through biotechnology without introducing transgenic components during GE.

For animals, gene delivery methods can be mechanical (e.g., microinjection, electroporation, or biolistic), chemical (e.g., lipid or nanoparticle carriers), or biological (e.g., viral or bacterial vectors). The most common method is the microinjection of genes into the pronuclei of zygotes, with an efficiency of approximately 100% (Yang and Wu 2018). The advantages of this method include high efficiency and fewer restrictions on the scale of delivery. The major methods for gene transfer into gametes include sperm-mediated gene transfer, somatic cell nuclear transfer (SCNT), and primordial germ cell migration (Shakweer et al. 2023). It is vital for animal breeding that the asexual reproduction by SCNT begins with genomes obtained from elite animals that can be engineered or edited. Improving livestock is an iterative process as further genetic modifications to incorporate desired traits are stacked on top of earlier alterations (Fan et al. 2023). Small non-coding RNAs, including small interfering RNA and microRNA, regulate genes at the post-transcriptional level through a variety of regulatory mechanisms and are widely used as alternatives for research involving experimental animals and cell lines. In the past few years, innovative nanoparticle-based delivery systems have been developed to introduce CRISPR/Cas9 constructs into animal cells and maximize their effectiveness (Givens et al. 2018; Mout et al. 2017).

Transgenic technology is also employed to introduce isolated and modified genes into the genomes of specific microorganisms, thereby enhancing their inherent traits or conferring new advantageous characteristics. Transgenic agricultural microbial technology has undergone multiple generations of advancement. These methods include microinjection, liposome-mediated methods, biolistic particle delivery methods, electroporation, and Agrobacterium-mediated transformation. Furthermore, with the advancement of GE technologies, particularly CRISPR/Cas, researchers can now delve deeper into the mechanisms of microbial action and modify microbial genomes with greater precision, enhancing the potential for the biocontrol of harmful microorganisms and optimizing the production, analysis, and utilization of agricultural microorganisms.

Synthetic biology, which integrates principles from biology, engineering, molecular biology, chemistry, and computational design to engineer and optimize novel biological systems or organisms with customized functions, has established a new paradigm in life science research, transitioning from the imitation of life to its design. Due to rapid advancements in DNA synthesis and artificial intelligence, the scale of synthetic genomics has evolved from the small genomes of microorganisms such as bacteriophages and mycoplasmas to those of eukaryotes such as yeast (*Saccharomyces cerevisiae*). The development of agricultural synthetic biology has accelerated, with a shift from designing individual gene components to integrating multiple components underlying complex traits. The field of synthetic biology in Chinese agriculture has achieved a series of significant breakthroughs. Human serum albumin (HSA) can be used clinically for the treatment of shock and burns and is extracted from plasma at high cost. The *HSA* gene was expressed in rice endosperm, successfully producing HSA and solving the problem of large-scale production, providing a safe and stable alternative for clinical use (He et al. 2011; Ning et al. 2008). Eight genes, namely *Phytoene synthase 1* (*ZmPSY1*) from the maize inbred line B73, *PaCrtI* from *Pantoea ananatis*, *β-carotene ketolase* (*CrBKT*) from *Chlamydomonas reinhardtii*, *HpCrtZ* from *Haematococcus pluvialis*, *XdCrtS* and *XdCrtR* from *Xanthophyllomyces dendrorhous*, and *β-carotene hydroxylase 1* (*AdCBFD1*) and *β-carotene ketolase 1* (*AdHBFD1*) from *Adonis aestivalis*, were used to create maize with significantly higher levels of the antioxidant astaxanthin in seed than previously reported. Astaxanthin-loaded microparticles prepared from this maize line by supercritical fluid extraction have shown high encapsulation efficiency and stability (Liu et al. 2021a). Chinese scientists recently identified the key enzymes (Taxane oxetanase 1 [TOT1] and Taxane 9α hydroxylase 1 [T9αH1]) in the biosynthetic pathway for the anti-cancer drug taxol, achieving the heterologous reconstruction of this important pathway in tobacco, marking China’s leading position in this field (Jiang et al. 2024). The discovery and application of these key genes have promoted the development of agricultural synthetic biology and provided new ideas and methods for the future.

### INNOVATIONS IN THE RESEARCH AND UTILIZATION OF AGRICULTURAL BIOTECHNOLOGY PRODUCTS IN CHINA

The Chinese government places great importance on the biosafety administration of agricultural biotechnology products and has developed laws and regulations spanning the entire spectrum of research, production, processing, importation, and labeling of GMOs and their associated products. This regulatory framework provides a scientific evaluation and supervision system to ensure safe cultivation, distribution, and consumption of these products (Gao et al. 2018b; Li et al. 2014; Liang et al. 2022). This framework governs biotechnology products (from animals, plants, and microorganisms) that are produced and utilized domestically and imported as raw materials for processing throughout their life cycles, from laboratory research on GMOs to restricted field testing, environmental release field testing, preproduction field testing, and biosafety certificate applications. Upon successful completion of safety evaluations for crops, a biosafety certificate can be obtained, representing the first step toward commercial planting.

**Fig. 1:**
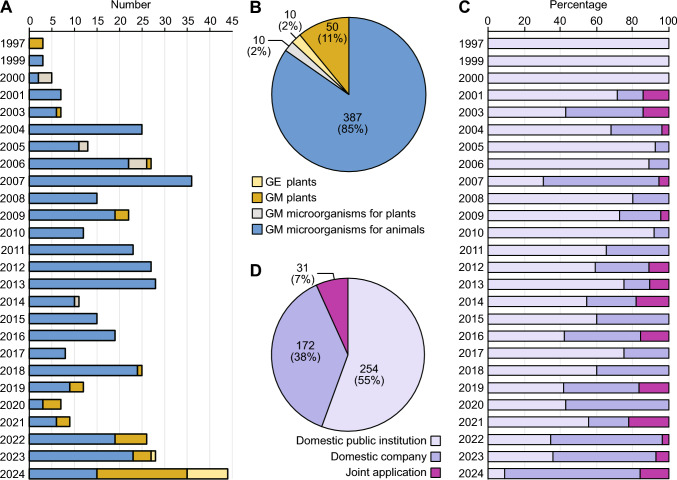
Approved agricultural GM and GE products with biosafety certificates in China. **A** Timeline of agricultural GM and GE product approvals for biosafety certificates in China. **B** Category profiles of agricultural GM and GE products that have obtained biosafety certificates since 1997. **C** Timeline of agricultural GM and GE products for each type of applicant profile. Percentages indicate the annual percentages of products that have obtained biosafety certificates. **D** Applicant profiles for agricultural GM and GE products that have obtained biosafety certificates since 1997. In **A** and **C**, the *y*-axis represents the year of the regulatory decision, and the *x*-axis represents the number (or percentage) of products. In **B** and **D**, the numbers in each section represent the number of approved GM and GE products, with their corresponding percentages in parentheses

In 1997, the Chinese government officially began accepting applications for the safety evaluation of agricultural GMOs and their products. According to the regulatory decision data from MARA, since 1997, biosafety certificates have been awarded for 457 GM and GE organisms and products (data collected until December 2024) (MARA 2024a) (Fig. 1). Overall, the number of approvals of bio-breeding products each year has fluctuated, likely due to national policies and market demand in a specific year. However, in recent years, the number of biosafety certificate approvals for agricultural GM and GE crops has rapidly increased. There has also been a substantial number of approvals for agricultural GM microorganisms (GMMs), predominantly those intended for animal use. Figure 1D shows the profiles of applicants: “domestic institutions” refers to public scientific research institutions and universities in China, and “joint” refers to collaborative efforts between domestic companies and scientific research institutions in shared initiatives for R&D and commercialization. Research institutions in China accounted for most approvals (55%), followed by domestic enterprises (38%) (Fig. 1D). Before 2010, domestic R&D on GM crops was exclusively controlled by domestic scientific research institutions. However, starting in 2011, domestic companies began to participate in the application process for biosafety evaluation; by 2020, they assumed a leadership role, with an increasing number of safety certifications obtained. This development aligns with the trajectory of scientific and technological progress. Specifically, during the transition from fundamental R&D to commercialization, when scientific research teams with mature technology are integrated into seed companies or collaborate through consortia, the research teams and companies play complementary roles. Therefore, strengthening their cooperation is crucial for enhancing the commercialization and innovative design of GM crops. Specific products are discussed in the next section.

### Product development for GM and GE crops

#### GM crops

The R&D and industrial application of GM crops in China are closely related to China’s market demands for agricultural products and national biosecurity and ecological protection strategies. Since the first safety evaluation in 1997, eight GM plants (cotton, papaya [*Carica papaya*], petunia [*Petunia* sp.], sweet pepper [*Capsicum annuum*], tomato, rice, maize, and soybean) have been approved for biosafety certificates in China. However, only Bt cotton (cotton varieties that are genetically engineered to carry the insecticidal gene from *B. thuringiensis*) and virus-resistant papaya are widely cultivated, as discussed below. Since the 1990s, cotton bollworm has become more prevalent in cotton fields, leading to heightened reliance on chemical pesticides and resistance to traditional pesticides among pests. Therefore, Guo et al. (2015) developed a GM Bt *GFM Cry1A* gene and bred China’s first generation of Bt transgenic cotton varieties, ‘GK12’ and ‘Jinmian 26’, in 1997. Ever since that milestone, GM cotton has been widely cultivated in China to control bollworm. In the 1960s, the outbreak of papaya ringspot virus (PRSV) in China could not be controlled by traditional breeding of disease-resistant varieties or managed with chemicals, posing a devastating threat to papaya yields. Chinese scientists investigated virus types affecting papaya varieties in China and developed ‘Huanong No. 1’, a virus-resistant papaya variety containing an RNA interference construct targeting the sequence encoding the PRSV replicase. This variety which obtained a biosafety certificate in Guangdong province in 2006. In 2009, two GM rice varieties, Huahui No. 1 and Bt Shanyou 63, which harbor the *Bt cry1A* gene, demonstrated high resistance to lepidopteran pests, and biosafety certificates were obtained for them. However, these varieties have not yet been certified by the national variety registration, and further commercial application is still pending variety validation.

**Fig. 2:**
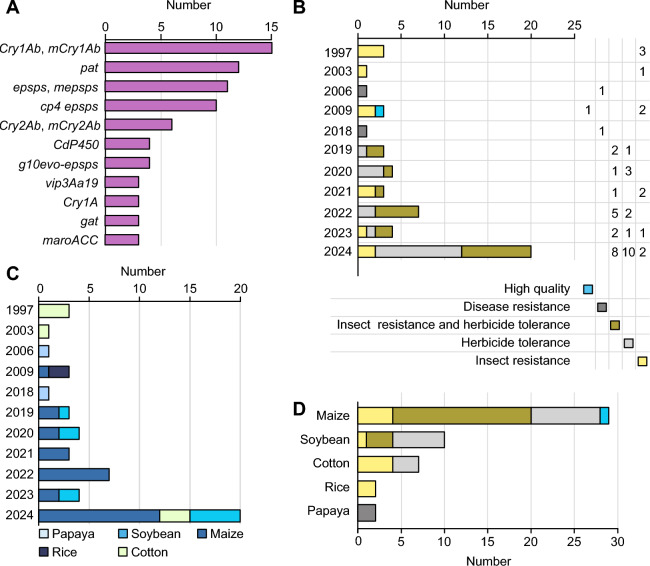
Approved agricultural GM plants with biosafety certificates in China. **A** Number of genes introduced into GM crops that have obtained biosafety certificates since 1997. Genes that have been utilized more than three times are listed. **B**, **C** Timeline of agricultural GM plant approvals for biosafety certificates in China per year with annual proportions by traits (**B**) and crop categories (**C**). The y-axis represents the year of the regulatory decision, and the x-axis represents the number of products. **D** Proportions of approved crops with biosafety certificates since 1997 listed by trait

Overall, 50 GM crop events have been initially approved for biosafety certificates (Fig. 2, Table 1). From 2001 to 2010, improvements in transgenic technology, coupled with the implementation of supportive national policies and industrial demands, stimulated enthusiasm among research institutions and businesses for the R&D of GMOs. Consequently, the number of approvals increased during this period (Fig. 2B), marking a new stage in the evolution of China’s GMO safety regulatory framework (Liang et al. 2022). The Chinese government has been increasing its investment in agricultural biotechnology. China’s bio-breeding program has achieved significant progress, introducing new crop varieties with traits including high yields, high quality, resistance to diseases and pests, drought tolerance, saline-alkaline tolerance, and efficient nutrient utilization. MARA issued biosafety certificates for production and application to insect-resistant (IR) and HT maize (‘DBN9936’ and ‘Ruifeng125’) and HT soybean (‘SHZD3201’) in December 2019. These lines provide area-wide pest management and significantly diminish the use of chemical pesticides (Yang et al. 2023).

**Table 1:** Agricultural GM and GE plant events recently approved for biosafety certificates in China

No.	Event name	Crop	Applicants	Introduced gene(s)/edited gene(s)	Commercial traits	First approval date and restricted region in China^a^
GM plants						
1	BFL4-2	Maize	Yuan Longping High-Tech Agriculture Co., Ltd. (Longping High-Tech), Biotechnology Research Institute, Chinese Academy of Agricultural Sciences	*cry1Ab*, *cry1F*, *cp4epsps*	IR, HT	2023-01-05 (Northern spring corn region) 2024-12-25 (Nationwide)
2	CC-2	Maize	China National Tree seed Corporation, China Agricultural University	*maroACC*	HT	2023-01-05 (Northern spring corn region) 2024–12-25 (Nationwide)
3	CAL16	Soybean	Hangzhou Ruifeng Bio-Tech Co Ltd	*cry1Ab::vip3Da*	IR	2023-01-05 (Southern soybean region) 2024-01-02 (Nationwide)
4	DBN8002	Soybean	Beijing Dabeinong Biotechnology Co., Ltd	*mvip3Aa*, *pat*	IR, HT	2023-04-21 (Huang-Huai-Hai summer soybean region)
5	DBN9936	Maize	Beijing Dabeinong Biotechnology Co., Ltd	*cry1Ab*, *epsps*	IR, HT	2019-12-2 (Northern spring corn region) 2024-01-02 (Nationwide)
6	Zheda Ruifeng 8 × nCX-1	Maize	Hangzhou Ruifeng Bio-Tech Co Ltd	*cry1Ab*, *cry2Ab*, *CdP450*, *cp4epsps*	IR, HT	2024-01-02 (Nationwide)
7	Ruifeng 125 × nCX-1	Maize	Hangzhou Ruifeng Bio-Tech Co Ltd	c*ry1Ab::cry2Aj*, *g10evo-epsps*, *CdP450*, *cp4epsp*	IR, HT	2024-01-02 (Nationwide)
8	Ruifeng 125	Maize	Hangzhou Ruifeng Bio-Tech Co Ltd	c*ry1Ab::cry2Aj*, *g10evo-epsps*	IR, HT	2019-12-02 (Northern spring corn region) 2024-01-02 (Northern spring corn region, Northwest corn region, Huang-Huai-Hai summer corn region)
9	LP026-2	Maize	Longping Biotechnology (Hainan) Co., Ltd	*cry2Ab*, *cry1Fa*, *cry1Ab*, *epsps*	IR, HT	2024-01-02 (Nationwide)
10	LW2-1	Maize	Longping Biotechnology (Hainan) Co., Ltd	*epsps*, *pat*	HT	2024-01-02 (Nationwide)
11	WYN17132	Maize	Wynca Zhejiang Xinan Chemical Industrial Group Co., Ltd	*am79epsps*	HT	2024-01-02 (Nationwide)
12	WYN041	Maize	Wynca Zhejiang Xinan Chemical Industrial Group Co., Ltd	*cry1Ab*, *am79epsps*	IR, HT	2024-01-02 (Nationwide)
13	WYN341GmC	Soybean	Wynca Zhejiang Xinan Chemical Industrial Group Co., Ltd	*cp4epsps*	HT	2024-01-02 (Nationwide)
14	WYN029GmA	Soybean	Wynca Zhejiang Xinan Chemical Industrial Group Co., Ltd	*mam79epsps*	HT	2024-01-02 (Nationwide)
15	Zhonghuang 6106	Soybean	Institute of Crop Sciences, Chinese Academy of Agricultural Sciences	*g2-epsps*	HT	2020-06-11(Northern spring soybean region) 2024-01-02 (Nationwide)
16	DBN9004	Soybean	Beijing Dabeinong Biotechnology Co., Ltd	*epsps*, *pat*	HT	2020-12-29 (Huang-Huai-Hai summer soybean region) 2024-01-02 (Nationwide)
17	BBL2-2	Maize	Origin Agritech Co., Ltd., Biotechnology Research Institute, Chinese Academy of Agricultural Sciences, Beijing Boaiyuanshang Biotechnology Co., Ltd	*cry1Ab, cry3Bb, cp4epsps*	IR, HT	2024-05-07 (Nationwide)
18	KJC017	Cotton	Cropedit Biotechnology Co., Ltd	*cp4epsps*	HT	2024-10-08 (Nationwide)
19	ND207	Maize	China National Tree Seed Group, China Agriculture University	*mcry1Ab, mcry2Ab*	IR	2021-12-17 (Northern spring corn region) 2024-12-25 (Nationwide)
20	DBN3601T	Maize	Beijing Dabeinong Biotechnology Co., Ltd	*cry1Ab, epsps, vip3Aa19, pat*	IR, HT	2021-12-17 (Southwest corn region) 2024-01-02 (Nationwide)
21	nCX-1	Maize	Hangzhou Ruifeng Bio-Tech Co Ltd	*CdP450*, *cp4epsps*	HT	2022-04-22 (Southern corn region) 2024-01-02 (Nationwide)
22	Bt11 × GA21	Maize	China National Seed Group	*cry1Ab, pat, mepsps*	IR, HT	2022-04-22 (Northern spring corn region) 2024-01-02 (Nationwide)
23	Bt11 × MIR162 × GA21	Maize	China National Seed Group	*cry1Ab, pat, vip3Aa20, mepsps*	IR, HT	2022-04-22 (Southwest corn region, Southern corn region) 2024-01-02 (Nationwide)
24	GA21	Maize	China National Seed Group	*mepsps*	HT	2022-04-22 (Northern spring corn region) 2024-01-02 (Nationwide)
25	DBN9858	Maize	Beijing Dabeinong Biotechnology Co., Ltd	*epsps, pat*	HT	2020-06-11 (Northern spring corn region) 2024-12-25 (Nationwide)
26	ND207 × CC-2	Maize	Beijing Liangyuan Biotechnology Co., Ltd	*mcry1Ab, mcry2Ab, maroACC*	IR, HT	2024-12-25 (Nationwide)
27	QY2569-42	Maize	Qingdao Qingyuan Seed Science Co., Ltd	*vip3Aa-k1, cry1Ab, pat*	IR, HT	2024-12-25 (Nationwide)
28	ZZM032	Maize	China National Seed Group	*bar, epsps*	HT	2024-12-25 (Nationwide)
29	MZIR260	Maize	China National Seed Group	*eCry1Gb.1Ig-03*	IR	2024-12-25 (Nationwide)
30	KJ1003	Maize	Cropedit Biotechnology Co., Ltd	*cry1A.105, cry2Ab2, vip3Aa19, cp4epsps*	IR, HT	2024-12-25 (Nationwide)
31	DBN9004 × DBN8002	Soybean	Beijing Dabeinong Biotechnology Co., Ltd	*epsps, pat, mvip3Aa, cp4epsps*	IR, HT	2024-12-25 (Nationwide)
32	DBN8205	Soybean	Beijing Dabeinong Biotechnology Co., Ltd	*cry1Ac, cry2Ab2, pat*	IR, HT	2024-12-25 (Nationwide)
33	XP-2	Soybean	Hangzhou Ruifeng Bio-Tech Co Ltd	*CdP450, cp4epsps*	HT	2024-12-25 (Nationwide)
34	GGK2	Cotton	Xinjiang Guoxin Seed Industry Co., Ltd., Biotechnology Research Institute, Chinese Academy of Agricultural Sciences	*gr79epsps*, *gat*	HT	2024-01-02 (Yellow River basin, Northwest inland region) 2024-12-25 (Nationwide)
GE plants						
35	AE15-18-1	Soybean	Shandong BellaGen Biotechnology Co., Ltd	*gmfad2-1a, gmfad2-1b*	High quality	2023-04-21 (Nationwide)
36	25T93-1	Soybean	Shandong BellaGen Biotechnology Co., Ltd	*GmELF3a*	Improved physiology	2024-01-02 (Nationwide)
37	P16	Soybean	Qi-Biodesign Biotechnology Co., Ltd	*GmFAD2-1A, GmFAD2-1B*	High quality	2024-01-02 (Nationwide)
38	179AC19-13-13	Maize	Shandong BellaGen Biotechnology Co., Ltd	*Br2*	High yield	2024-05-07 (Nationwide)
39	MLO-KNRNP	Wheat	Suzhou Qi-Biodesign Biotechnology Co., Ltd., Institute of Genetics and Developmental Biology, Chinese Academy	*TaMLO-A1, TaMLO-B1, TaMLO-D1, TaMLOX*	Disease resistance	2024-05-07 (Nationwide)
40	QH64112	Soybean	Suzhou Qi-Biodesign Biotechnology Co., Ltd., Beijing Qi-Biodesign Biotechnology Co., Ltd	*GmLn*	High yield	2024-12-25 (Nationwide)
41	E001SYFT	Soybean	China National Seed Group	*GmE1, GmE1Lb*	Improved physiology	2024-12-25 (Nationwide)
42	KN-NL4-2	Maize	Wimi Biotechnology Co., Ltd., Huazhong Agricultural University	*ZmNL4*	High yield	2024-12-25 (Nationwide)
43	118-9-15	Rice	Suzhou Qi-Biodesign Biotechnology Co., Ltd., Jiangsu Academy of Agricultural Science, Beijing Qi-Biodesign Biotechnology Co., Ltd	*Wx*	High quality	2024-12-25 (Nationwide)
44	TaALS-4	Wheat	Suzhou Qi-Biodesign Biotechnology Co., Ltd., Institute of Genetics and Developmental Biology, Chinese Academy	*TaALS*	HT	2024-12-25 (Nationwide)

Crops and biosafety certificates approved from January 5th, 2023 to December 25th, 2024; approvals before 2023 are listed in Liang et al. (2022)

*IR* insect resistance, *HT* herbicide tolerance

^a^The GMO biosafety certificates restricted the planting area of each GMO. Before 2023, applicants can apply biosafety certificates for a certain cultivation area (e.g., Northern spring corn region). Since the revised regulations have been carried out by MARA in 2023, applicants were encouraged to apply a nationwide biosafety certificate that covered almost all cultivation area of each GMO

Fall armyworm (*Spodoptera frugiperda*), an insect pest native to the Americas, invaded China in December 2018 and has been spreading rapidly attributed to its high rates of reproduction and migration (Sun et al. 2021; Wang et al. 2019; Zhu et al. 2022). However, due to the evolution of chemical resistance in populations of *S. frugiperda* and the lack of systematic prevention strategies, traditional insecticides have gradually lost their ability to control this insect pest. Furthermore, plant lines with a single *Bt-Cry1Ab* gene were shown to have lower insecticidal effects against fall armyworm compared to those with dual *Bt-Cry1ab* + *Vip3Aa* genes (Zhang and Wu 2019; Zhang et al. 2024; Zhao et al. 2023; Zhou et al. 2023). Based on the biological characteristics and occurrence patterns of fall armyworm, and drawing from the experience gained in managing cotton bollworm and other major agricultural pests in China, various strategies for the prevention and control of fall armyworm have been proposed (Li et al. 2022a; Wu 2020; Zhou et al. 2021).

The number of approvals reached an all-time high in 2024. However, the number of approvals was lower in 2021 and 2023, which may be attributed to two factors: the increased interest in multi-gene stacking and the change in focus from GM to GE crops. GM maize, soybean, and cotton predominate, accounting for 90% of all approved GM crops (Fig. 2C). IR and HT GM maize, HT soybean, and IR cotton are the most developed GM traits (Fig. 2D). As shown in Fig. 2A, the major genes that have been introduced into GM crops have been utilized in bio-breeding for several years, such as *epsps*, phosphinothricin acetyltransferase gene (*pat*), *cry1Ab*, and *cry2Ab*. Technological advancements and market competition have progressively led to a shift from single-trait and single-gene events to multi-gene stacked traits. Since 2019, there has been a surge in approvals of new lines featuring IR or HT traits, which were developed by introducing fusion genes or modified genes. For example, researchers have engineered GM crops with resistance to alternative herbicides. The *CdP450* gene, developed originally by Chinese scientists, confers tolerance to multiple herbicides and has received approval to enhance resistance against the herbicides 2,4-dichlorophenoxyacetic acid (2,4-D) and flazasulfuron in GM maize. At the same time, some traditional and single-gene IR lines that have been used for many years have been slowly phased out. These advancements offer improved resistance strategies for commercial cultivation (Yang et al. 2024) (Fig. 2B).

To improve bio-breeding products and meet current market demands and national strategies, MARA encourages innovation while avoiding low-level and homogenized research and products (MARA 2021). A series of new IR and HT maize and soybean varieties have obtained biosafety certificates for production and utilization. Companies such as Beijing Dabeinong Biotechnology, Longping Biology, and Hangzhou Ruifeng are leading the charge, developing new traits and genes: MARA issued domestic biosafety certificates for the production and utilization of IR soybean lines ‘CAL16’ and ‘DBN8002’ in 2023 and the HT cotton lines ‘GGK2’ and ‘KJC017’ in 2024 (Table 1). With the issue of GE regulations, developers have intensified their R&D efforts on new bio-breeding materials and traits, resulting in a notable decrease in approvals of GM crops in 2023 along with an increase in approvals of GE crops in 2024.

China has completed the entire approval framework for the commercial planting of GM maize and soybean and is poised to undergo substantial commercial planting. Since 2022, MARA has released several regulations and standards to help domestic developers obtain approval for GM soybean and maize varieties, further accelerating the commercial cultivation of these GM crops in China. Up to November 2024, MARA has approved 64 GM maize varieties and 17 GM soybean varieties (Announcement No. 732 and No. 830 of the MARA), and 39 companies have obtained licenses for the production and operation of GM maize and GM soybean seed (Announcement No. 739 and No. 842 of the MARA). From 2021 to 2023, pilot industrialization projects for GM soybean and maize were initiated in China. Planting GM maize and soybean has decreased the need for pest control measures and weed management practices, yielding notable improvements in yield efficiency while having remarkable ecological benefits; this result marks a historic step forward in efforts to test and industrialize GM soybean and maize (Sun et al. 2024; Xinhua 2023). To prepare for the further commercialization of GM plants, MARA also released the “Requirements for the Registration of Target Herbicides Used on Genetically Modified Herbicide-Tolerant Crops,” and approved the extended application of certain glyphosate formulations to glyphosate-resistant maize and soybean (MARA 2022a, 2024b).

#### GE crops

GE technology, an emerging approach for bio-breeding, has been firmly embraced by the Chinese government and has become a prominent focus of regulation making and R&D investment. In 2016, a series of initiatives was undertaken focusing on the development and industrialization of GE technology, including the 13th Five-Year Plan National Science and Technology Innovation Plan, the 13th Five-Year Plan Biotechnology Innovation Special Plan, the 13th Five-Year Plan Biological Industry Development Plan, and the 13th Five-Year Plan National Strategic Emerging Industry Development Plan (NDRC 2017). Furthermore, both the 14th Five-Year Plan for National Economic and Social Development of the People’s Republic of China in 2023 and the Outline of the 2035 Vision Goals explicitly designate GE technology and biotechnology as frontier fields within science and technology.

Safety evaluations of GE crops began later in China than in other countries, but now progress is being made in line with global progress. China has been gradually optimizing the approval procedure to better accommodate national conditions. Unlike many other countries, China classifies GE plants under its GMOs regulatory framework but has established a simplified version of the GMO regulatory framework for GE plants exhibiting no foreign DNA integration (USDA 2022; Vora et al. 2023). In January 2022, MARA released “Guidelines for Safety Evaluation of Gene-edited Plants for Agricultural Use (Trial),” followed by “Evaluation Rules for Gene-edited Plants for Agricultural Use (Trial)” on April 28, 2023 (MARA 2022b, 2023b), significantly accelerating the breeding of GE crops. Soybean variety ‘AE15-18–1’, an improved-quality variety harboring mutant *GmFAD2-1a* and *GmFAD2-1b* genes encoding fatty acid desaturase 2–1, was developed by Shandong Shunfeng Biotechnology Co., Ltd. This variety obtained a production and application biosafety certificate, becoming the first approved GE crop in China, laying the foundation for further commercial development of GE crops in China (Table 1).

Most GE crops are designed to improve quality, physiological, yield, and abiotic/biotic stress-resistance traits. These types of products closely resemble outcomes from natural mutation breeding; therefore, their safety risk level is low, making them more likely to receive approval for biosafety certificates. In 2024, MARA granted its first biosafety certificates to GE powdery mildew-resistant wheat, GE maize with enhanced yield, and HT wheat, representing a significant milestone for GE crops that are as important to national food security as IR and HT maize.

### GM and GE animals

The use of biotechnology in animals has always been more difficult compared to plants and microorganisms due to the biological and functional complexity of animals. On a global scale, persistent regulatory uncertainties frequently emerge as a primary constraint on the commercialization of animal biotechnology (Fan et al. 2022b). China was a global pioneer in research on transgenic animals. In 1983, Zhu et al. (1985) successfully introduced a growth hormone gene into fertilized crucian carp (*Carassius carassius*) eggs, resulting in transgenic fish with accelerated growth. Over the past 40 years, with the support of several major national projects, China has made significant advancements in GM animal research. In 1999, China bred the first transgenic cow containing the *HSA* gene. The levels of human lactoferrin, human alpha whey protein, and human lysozyme in the milk of transgenic cattle bred from 2003 to 2006 reached high levels. In 2003, for the first time, three foreign genes were introduced into the same cow by a research group at China Agricultural University, underlining the maturity of China’s production technology system for cattle cloned from transgenic somatic cells. In 2006, the world’s first GM cow resistant to mad cow disease was born. Furthermore, in July 2010, the genetic modification of beef cattle resulted in higher meat production and improved meat quality compared to non-GM breeds (Huang et al. 2011). Transgenic mice (*Mus musculus*), rabbits (*Oryctolagus cuniculus*), goats (*Capra hircus*), and pigs were also developed to help scientists solve fundamental research questions (Zou et al. 2021).

The emergence of GE technologies such as transcription activator-like effector nucleases, zinc finger nucleases, and CRISPR/Cas9 has propelled agricultural animal and aquaculture breeding into the fast lane in the current decade. China has conducted extensive research on GE animals in areas such as production, disease resistance, and nutrition. GE animals are expected to play an increasingly important role in sustainable agriculture and the protection of biodiversity. However, the approval of biosafety certificates for the production and application of agricultural GM and GE animals has been slow (Fan et al. 2021). Therefore, until 2024, no agricultural GM or GE animals have been approved in China for a biosafety certificate.

### GM and GE microorganisms

Microbes have been utilized to enhance agriculture, facilitate food production, and combat diseases. Similar to other regulated agricultural GMOs, the term GMMs refers to recombinant microorganisms and their products employed in animal or plant-based agricultural production or processing that were generated through genetic engineering techniques that alter genome composition. According to China’s regulatory framework, GMMs for agriculture are primarily categorized into GMMs for animal or plant applications. Acquiring safety certifications merely represents an initial step toward commercializing these products, as they must undergo additional evaluations by various regulatory and administration departments prior to commercialization. For example, GMMs for animal use, especially GMMs producing active substances, must obtain biosafety certificates as well as registration certificates for veterinary drug use before their commercialization. However, according to Regulations on Administration of Veterinary Drugs (Order No. 404 of the State Council), when registering veterinary biological products that do not contain active substances from GMMs, documents on the safety evaluation of GMOs do not need to be submitted when applying for clinical trials.

**Fig. 3:**
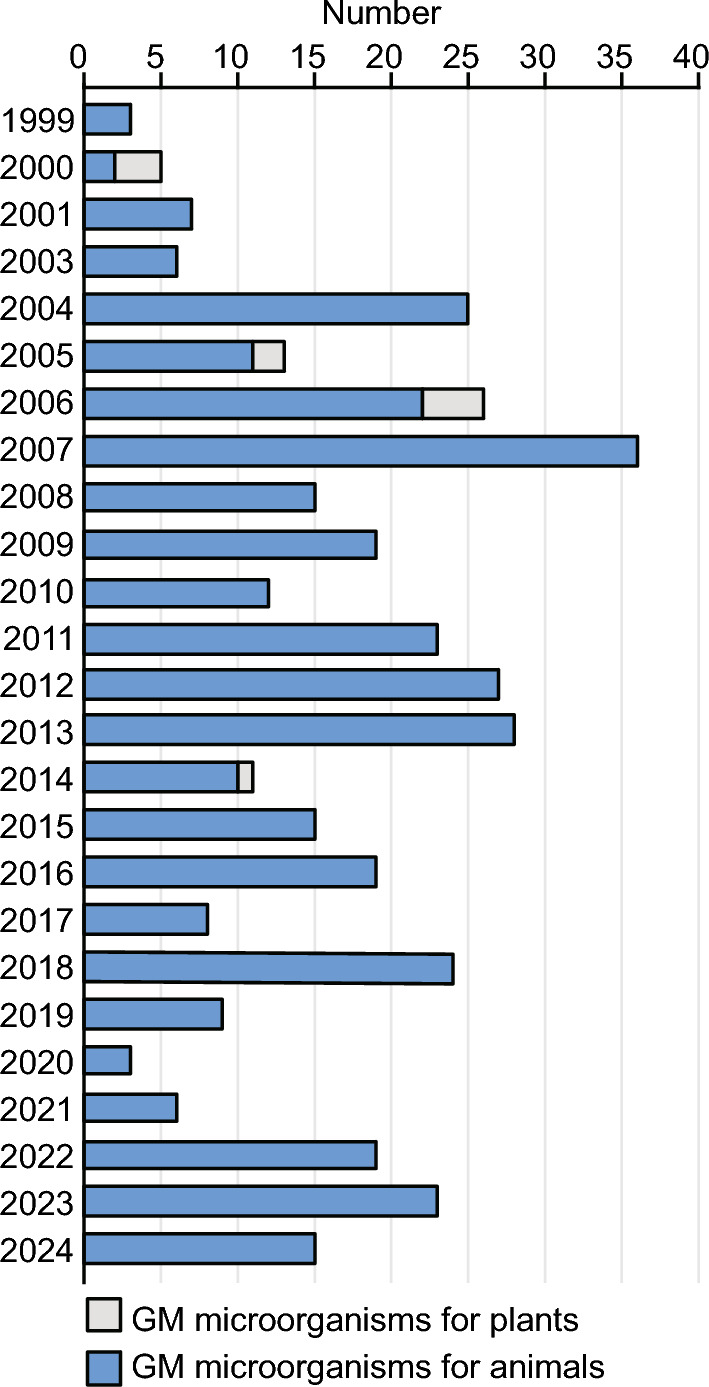
The timeline for approval of agricultural GMMs with biosafety certificates in China. The y-axis represents the year of the regulatory decision, and the x-axis represents the number of products

GE technologies such as CRISPR/Cas9 have shown tremendous potential for the development of microbial vaccines and biofertilizers. GE microbial vaccines, facilitating precise alterations of pathogen genomes, have improved efficacy and safety. GE enables customized antigen optimization, enhanced vector design, and a more profound understanding of the influence of host genes on vaccine responses, ultimately advancing the processes of vaccine development and manufacturing and playing a key role in animal health management (Bisht et al. 2024). Transgenic microorganisms have shown great promise as plant growth-promoting agents and biofertilizers by improving soil fertility and enhancing plant growth. Since the first approval in 1999, 397 GMMs have been approved for biosafety certificates (Fig. 3). Among these, 387 are for animal use, and 10 are for plant use, since China issued ‘Guidelines for the Safety Evaluation of Genetically Modified Microorganisms for Animal Use’ in 2010 but has yet to release similar guidelines regarding GMMs for plant use.

#### GMMs for animal use

**Fig. 4:**
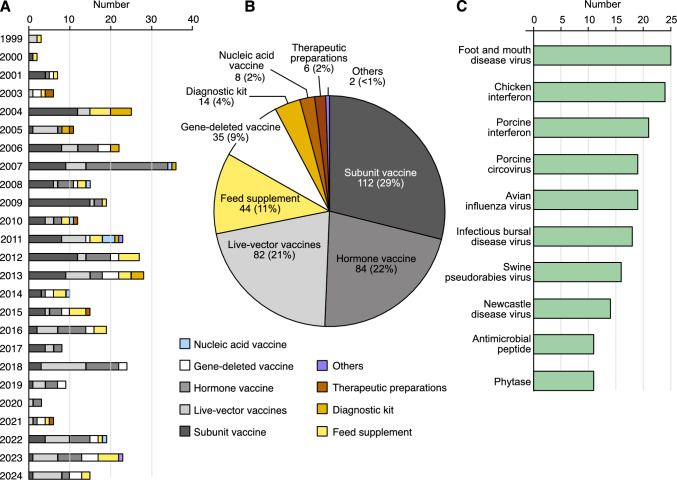
Approved agricultural GMMs for animal use with biosafety certificates in China. **A** Timeline of approval of agricultural GMMs for animal use in China. The *y*-axis represents the year of the regulatory decision, and the *x*-axis represents the number of products. B, C Categories (**B**) and top 10 traits profiles (**C**) of agricultural GMM products for animal use that have obtained biosafety certificates since 1999. Numbers in each section represent the number of approved GMM products and their corresponding percentage in parentheses

According to the regulatory framework, GMMs for animal use are categorized into those genetically engineered to produce subunit vaccines (containing single or multiple viral proteins), recombinant live-attenuated vaccines, gene-deleted vaccines, nucleic acid vaccines, hormone vaccines and therapeutic preparations, GMMs for feed, and those genetically engineered to produce antigens for diagnostic kits. Among these, approvals of GMMs have mainly focused on the production of subunit vaccines, hormone vaccines and live-attenuated vaccines (Fig. 4A and B), likely reflecting market demand related to livestock breeding and prevalent epidemic diseases. The overall approval landscape aligns with national consumer preferences for chicken and pork. China’s strategy for the prevention and control of major acute animal infectious diseases involves vaccination (as well as culling), which also serves as the driving force behind R&D for new animal vaccine technologies. Vaccine products account for approximately 83% of all approvals (Fig. 4B), predominantly targeting pathogens such as avian influenza virus, Newcastle disease virus, bursal virus, swine circovirus, foot-and-mouth disease virus, and swine fever virus (Fig. 4C).

##### Animal vaccines

R&D of genetically engineered animal vaccines in China began in the 1990s. Several types of genetically engineered vaccines (including subunit vaccines, live-attenuated viral vector-based vaccines, gene-deleted vaccines, and DNA vaccines) have been industrialized and are widely used in China.

Gene-deleted vaccines: Gene-deleted vaccines are produced by deleting virulence genes from a virulent virus. A *TK/gE/gI* triple gene-deleted pseudorabies virus vaccine strain and a *TK/gG* double gene-deleted pseudorabies virus vaccine strain were successfully developed in 2000. Trials demonstrated that these strains are safe for various pigs and other animals. Having obtained new veterinary drug certificates, these strains have been used in China for over a decade. In response to new variants of pseudorabies virus emerging in recent years in China, double gene-deleted *gE/gI* (Tong et al. 2016) and triple gene-deleted *TK/gE/gI* (Zhang et al. 2015) vaccine strains were rapidly developed using CRISPR/Cas9 alongside homologous recombination technology; these strains are currently in use, with significant preventive effects. Chen et al. (2020) developed a seven gene-deleted African swine fever virus live-attenuated gene-deleted vaccine (HLJ/18-7GD), which showed good immunoprotective efficacy in clinical trials. Over the past 2 decades, various gene-deleted attenuated live vaccines against *Brucella* bacteria have been developed (Li et al. 2015, 2018; Liu et al. 2020b; Wang et al. 2014). Xu et al. (2021a) constructed three different gene-deleted *Brucella* vaccine strains (BAO711-△cspA, M5-90-△bp26, and A19-△VirB12) based on strains BAO711, M5-90, and A19, respectively. These three vaccine strains have obtained new veterinary drug registration certificates and have begun commercial production and utilization in China.

Live viral vector vaccines: given the continuous development and expansion of animal husbandry practices, effective prevention and control measures against animal diseases are crucial. Vaccination remains the most efficient, cost-effective approach for disease prevention and control. Among gene manipulation techniques employed in vaccine construction (Fig. 4A), there has been a gradual decline in approved subunit vaccines, which are being replaced by live vector vaccines. Genetic engineering is employed to introduce genes encoding antigens against pathogens into live vectors (Draper and Heeney 2010). This technique has several advantages, including the excellent safety of inactivated vaccines, good immune effects, low cost, and lowering the risk of atavism of virulence. Multiple antigen genes from different pathogens can be inserted into a vector and expressed at the same time to prevent multiple diseases (Jorge and Dellagostin 2017). To date, only recombinant fowlpox virus-based vaccines expressing either infectious laryngotracheitis virus gB protein (Tong et al. 2001) or highly pathogenic avian influenza virus HA protein developed in China were granted new veterinary drug certificates (in 2005) and are currently in commercial use (Qiao et al. 2009). Moreover, the vector vaccine against the highly pathogenic avian influenza duck plague virus obtained a new veterinary medicine registration certificate (Liu et al. 2011), making it the first duck plague virus vector vaccine to proceed toward commercial use worldwide. Although vaccines based on RNA virus vectors have displayed effective immunoprotective effects on their target animals or animal models, very few have advanced to clinical or commercial use. The one exception is a recombinant Newcastle disease virus vector vaccine expressing the HA protein of highly pathogenic avian influenza virus, which was approved for industrial use in China in 2006 (Chen and Bu 2009). A recombinant PRRS virus vector vaccine expressing the classical swine fever virus E2 protein exhibited 100% protective efficacy against virulent classical swine fever virus without interference from maternal antibodies and is poised to enter clinical trials (Gao et al. 2018a, 2020).

Subunit vaccines: several subunit vaccines have been approved for commercial use, including the classical swine fever virus E2 protein subunit vaccine expressed in a baculovirus system, the infectious bursal disease virus VP2 protein subunit vaccine produced in *Escherichia coli*, the foot-and-mouth disease virus capsid protein VLP vaccine produced in *E. coli* (Teng et al. 2021), and the porcine circovirus type 2 Cap protein subunit vaccine expressed in a baculovirus system.

Nucleic acid vaccines: compared to traditional vaccines, DNA vaccines are simpler to manufacture, faster to produce, more cost-effective, easier to subject to quality control, provide prolonged immunity, exhibit thermal stability, and are convenient to store and transport. However, to date, few DNA vaccines have been approved. In 2018, China approved its first DNA vaccine, which is designed to prevent highly pathogenic avian influenza (Jiang et al. 2007).

##### Feed additives

GMMs play crucial roles in various aspects of animal health and production. In addition to vaccines, GMMs are utilized to produce growth hormones and other bioactive factors that stimulate animal growth, resulting in increased meat, egg, and milk production (Choi and Lee 2023). GMMs are also used for the development of therapeutic proteins (such as interferon and insulin) to treat a range of animal diseases (Lobocka et al. 2021). Most approved agricultural GMMs are used in animal husbandry. For example, GMM probiotics are used to enhance intestinal health, improve digestive system function, and boost feed efficiency and growth performance in animals (Fig. 4B). GMM-based feed additives are primarily used in the synthesis of chicken interferon and pig interferon products and as feed enzymes. Microbial fermentation technology is ideal for producing feed due to the rapid reproduction rates, high metabolic efficiencies, and large product yields of microorganisms. *E. coli*, *Bacillus*, budding yeast, *Pichia pastoris*, and *Aspergillus* are frequently used for the heterologous production of xylanase (Wang et al. 2024), β-mannanase (Liu et al. 2021b), phytase (Helian et al. 2020), and other products.

#### GMMs for plant use

Studies on GMMs for plant use mainly focus on insecticidal microorganisms and biofertilizers. *Bt* is currently the most widely employed insecticidal microorganism globally (Gomis-Cebolla and Berry 2023; Li et al. 2022b). Transgenic technology enhances insecticidal activity, broadens the spectrum of pest control, and allows for the introduction of *Bt* genes into other host strains to confer insecticidal properties. Biofertilizers also play an important role in sustainable agriculture by improving soil fertility and plant growth while reducing the dependency on chemical fertilizers. However, developing GMMs for plant use involves higher costs associated with R&D, field trials, environmental impact assessments, safety verification, and registration compared to naturally isolated strains. Indeed, although *Bt*-derived strains are the most successful commercialized GMM product, products derived from natural strains dominate the microbial pesticide market. Advanced genetic engineering tools like CRISPR and synthetic biology demand costly infrastructure and expertise. These factors have dampened the enthusiasm of R&D entities, resulting in a limited number of biosafety certificates for GMMs for plant use. Consequently, industrial applications of such GMMs remain scarce.

##### Growth-promoting agents

GMMs affect various aspects of plant development and agricultural practices. Certain microorganisms enhance nutrient uptake and stimulate growth by producing essential plant hormones, such as gibberellins and cytokinins. For instance, specific fungi have been engineered to produce enzymes or synthesize organic acids that promote plant growth (Singh et al. 2022). Furthermore, some GMMs effectively prevent and control plant diseases through the incorporation of genes conferring resistance to or imposing toxic effects on target species. For example, biopesticides with targeted effects on pests have been produced based on *Bt* (Li and Liu 2015). The *Bt*-engineered strain G033A represents the first *Bt*-engineered product in China to receive pesticide registration, addressing the need for effective control of coleopteran pests in China. In 2006, this *Bt* strain received a biosafety certificate for nationwide industrial application and promotion. In 2023, it was rated as a major new product in China’s agricultural sector.

The use of microorganisms to produce proteins that can improve crop immunity has a wide range of prospective applications. The plant immune-activating protein *Verticillium dahliae*-secreted Asp f2-like developed by China Agricultural University has been commercialized, and it can effectively stimulate the autoimmunity of crops, improve the physiological and biochemical functions of crops, and improve the ability to cope with pathogens and extreme environments (Ma et al. 2021). *Pseudomonas* ssp. is a well-known plant growth-promoting rhizobacteria for their ability to fix nitrogen and biocontrol of soil pathogens. Zhang et al. (1998) conducted recombineering, a genetic engineering technique that uses homologous recombination systems (e.g., the λ Red system in *E. coli*) to precisely modify DNA in vivo, to generate several plant growth-promoting GMMs (Li et al. 2023). For example, an entire nitrogenase genomic island from *P. stutzeri* DSM4166 was introduced into the genome of *P. protegens* CHA0 and Pf-5 strains, which have both nitrogen-fixing and bactericidal activities. The application of this GM strain has led to significant improvements in both soil properties and crop quality (Yu et al. 2018; Jing et al. 2020). Furthermore, a combination of *Aspergillus niger* with GM *Pseudomonas* ssp. has been demonstrated to improve both production and quality of lettuce (*Lactuca sativa*) and cucumber (*Cucumis sativus*) planted on salinized soil (Ni et al. 2023).

##### Biofertilizers

GMMs contribute to soil remediation by degradation of harmful substances like pesticide residues, thereby mitigating environmental pollution. The overuse of chemical fertilizers has led to the deterioration of arable lands, polluted farmland environments, and decreased agricultural product quality, thus hindering sustainable agriculture. To address these challenges, the use of biofertilizers increased in the 1990s, leading to significant progress over the past 30 years. The microbes *Rhizobium*, arbuscular mycorrhizal fungi, *Bacillus*, *Pseudomonas*, and *Trichoderma* are widely used as biofertilizers. Specifically, modified white rot fungi and actinomycetes decompose toxic organic compounds in the soil, thereby improving overall soil quality (He et al. 2021). These microorganisms can be utilized as biofertilizers to supply essential nutrients required for plant growth. For instance, *Rhizobium* is capable of nitrogen fixation, providing leguminous plants with a sustainable nitrogen source (Ali et al. 2020). New strains of GM or transgenic microorganisms can be developed using GM/GE techniques to produce biofertilizers. Microbe-plant communication has been studied to strengthen the regulation of microbial signaling pathways for plant growth. For example, Boo et al. (2024) developed modular interkingdom communication channels, by introducing a “sender device” into *Pseudomonas putida* and *Klebsiella pneumoniae* that produces the small molecule *p*-coumaroyl-homoserine lactone (pC-HSL), which triggers a pC-HSL-specific promoter “receiver device” in the plant to generate a transcriptional output.

## Discussion, conclusion and prospects

Modern agricultural biotechnology breeding includes the application of GM and GE technology, genome-wide selection, synthetic biology, and other approaches to achieve efficient, precise, and targeted genetic improvement and for breeding new varieties of plants, animals, and microorganisms. The cultivation of staple crops in China, including maize, rice, wheat, soybean, rapeseed (*Brassica napus*), and cotton, relies on seeds that have been meticulously bred within China, ensuring the security of the food supply chain and the reliable provision of essential agricultural commodities. However, compared to other advanced nations, data from 2020 reveal that China’s the average yield of maize and soybean per unit area is less than 60% that of the United States, with this discrepancy increasing between 2010 and 2020 (Zhang 2022). Consequently, promoting the industrialization of biotechnology breeding is inevitable for the development of the modern breeding market in China and to ensure national food security and a reliable supply of important agricultural products. China and the international community require GMOs more than ever before.

It is projected that 581 million mu (15 mu = 1 hectare) of GM maize and 139 million mu of GM soybean will be planted in China if the rate of GM crop utilization reaches 90% in 5 years (Sun et al. 2024). To accelerate commercialization of GM and GE organisms and ensure the biosafety of bio-breeding products, the Chinese government has recently begun to issue a series of policies to stimulate R&D on bio-breeding and biosafety. In 2022, the National Development and Reform Commission (NDRC) issued the “14th Five-Year Plan for Bioeconomic Development,” which is China’s first 5-year plan for the bioeconomy. This plan promotes the development of biotechnology products, including maize and soybean, and aims to accelerate breakthroughs and achieve scientific and technological self-reliance in key areas of bioeconomic development. MARA released the “Implementation Plan Outlining the Key Initiatives for Comprehensively Advancing Rural Vitalization of 2023,” which emphasizes the full execution of the National Project on Bio-breeding for Agricultural Crops, ensuring substantial progress in collaborative R&D efforts related to breeding and genetic improvement programs for livestock. It is also essential to accelerate commercialization within the bio-breeding sector and to expand trial applications for GM maize and soybean. Finally, the 2024 China No. 1 Central Document proposes to “accelerate the expansion and industrialization of biological breeding.” Therefore, China aims to accelerate the selection, promotion, and production of urgently needed independent superior crop varieties while enhancing research on key and core technologies related to germplasm innovation.

Despite the current policy support, China may need to undertake the following strategic actions: (a) strengthen policy guidance and implement long-term programs for scientific and technological development to promote the capacity of R&D in biotechnology; (b) foster confidence in the development and application of GM and GE organisms to attract businesses investment to this industry; (c) strengthen the development of the biosafety regulatory system for GM and GE organisms and expedite the regulatory procedures; and (d) develop and implement a well-targeted biotechnology public engagement initiatives to address misinformation propagated from anti-biotechnology groups (Li et al. 2020c).

### Plant biotechnology products

China is poised to initiate large-scale commercialization of GM maize and soybean within the coming decade. Key considerations include the following: (a) learning from international GM crop cultivation practices to develop resistance management strategies for pests and weeds, encompassing planting strategies and stacked gene pyramiding for new traits, as well as resistance monitoring; (b) ensuring the coexistence of GM and non-GM products to meet diverse consumer preferences and maintain market diversity through policies that balance biosafety, development, and regulatory costs; (c) developing new biotechnology products with traits designed to address evolving agricultural challenges by leveraging multi-omics approaches and big data analytics, integrating GE with transgenic technologies to enhance crop performance and sustainability; and (d) expanding the application scenarios of GM and GE plants, such as using plants as bioreactors to provide a variety of nutrient-enhanced food components (e.g., high-amylose starch) or medical raw materials (e.g., HSA and vaccines).

Plant-derived molecular drugs offer numerous advantages. For instance, approximately 10 g of recombinant HSA can be produced from 1 kg of rice seed. Once large-scale production has been achieved, the cost of recombinant HSA will be significantly lower than that of plasma-derived HSA (He et al. 2011). In another study, a coronavirus-like particle vaccine was developed using plant molecular farming platforms, which successfully generated several viral vaccines with significant immunogenicity and efficacy against coronavirus disease 2019 (Covid-19) (Hager et al. 2022), all of which have advanced to clinical phase III trials. With ongoing technological advancements—including the optimization of glycosylation modification systems and endoplasmic reticulum protein quality control mechanisms—as well as integration with GE techniques and synthetic biology, there is potential to achieve a humanized glycosylation pipeline in plant bioreactors for heterologous protein production. This approach could enhance both the quality and yield of plant-derived therapeutic proteins while significantly contributing to pharmaceutical development. The final consideration will be establishing a robust intellectual property system to support R&D companies facing high investment costs and long payback periods, thereby ensuring sustainable development through equitable benefit distribution.

### Animal biotechnology products

The use of transgenic technology in animals has a history spanning over 40 years and has significantly contributed to basic research on animal gene function, medicine, and agriculture. To date, the only transgenic animal approved for food consumption is salmon (*Salmo salar*) in the United States, although three other transgenic animals (goats, rabbits, and chickens) have been approved solely for medical purposes related to their expressed products rather than enhancing the production traits of the animals themselves. Globally, except for the European Union, most countries maintain lenient regulations regarding new breeding technologies and are contemplating approval of GE livestock as a component of breeding programs aimed at producing food for human consumption.

To date, China has not issued biosafety certificates for any agricultural GM or GE animals (Huang et al. 2022). China lacks a regulatory framework specifically to GE animals, but China’s MARA currently regulates GE animals in a manner similar to GMOs. In June 2023, MARA began to develop Guidelines for the Safety Evaluation of Gene-Edited Animals for Agricultural Use, and feedback was solicited from relevant stakeholders and experts. The successful commercialization of GM crops in China has provided favorable conditions for the steady progress of GM animal R&D (Huang et al. 2022). The approval of transgenic salmon in the United States served as a valuable reference for the global commercialization of GM and GE animals in various economies (Fletcher et al. 2004; USFDA 2015). The safety evaluation procedure of GE animals should use case-by-case analysis, streamline data requirements, and optimize reporting procedures. Furthermore, it is essential to begin research and policy formulation related to the commercial application process following safety evaluations to facilitate the commercialization of GE animals by providing direction and fostering a supportive regulatory environment.

### Microorganism biotechnology products

GMMs are extensively used in agriculture, but their functional classification is inherently complex. In China, current biosafety certificates for GMMs primarily pertain to GMMs for animal use, especially vaccines and feed additives. Therefore, to ensure safety and efficacy: (a) future animal vaccines should be designed that retain the advantages of existing vaccines (including traditional and genetically engineered vaccines) while overcoming their disadvantages; (b) mainstream approaches should be employed for the future development of animal vaccines, such as multi-epitope peptide particulate vaccines, personalized vaccines, specific immunostimulants, and needle-free intradermal immunization (particularly suitable for swine vaccines); (c) more ecologically sustainable feed should be generated in light of the increasing emphasis on environmental conservation worldwide; and (d) the customization of animal feed products should be prioritized, since the nutritional requirements of animals vary based on species, growth stage, health status, and other factors. This milestone could be achieved by precisely controlling the types and quantities of microorganisms in the feed and creating tailored solutions for specific farming objectives and conditions.

Over the past 30 years, the development and utilization of biofertilizers have contributed to agricultural sustainability and productivity. As the demand for sustainable food production increases, along with the need to conserve resources and protect the environment, biofertilizers are poised to play even more important roles in agricultural production. In contrast to the rapid development of chemical fertilizers, biofertilizers still have many issues to address: (a) there is an urgent need for microbial breeding technologies tailored for biofertilizer production; (b) with the development of synthetic microbial communities (SynComs), understanding the microbial interactions among SynCom members is crucial for enhancing the stability of multi-species biofertilizers (Gomez-Godinez et al. 2021); and (c) innovative biofertilizers designed to enhance plant resistance to abiotic stress are urgently needed. These newly developed products should focus on improving crop tolerance to salinity, drought, and high temperatures. Maximizing the effectiveness of biofertilizers requires a comprehensive understanding of interactions among the soil, biofertilizers, and plants.

### Synthetic biology products

Synthetic biology is a multidisciplinary domain that integrates biotechnology, engineering, and software disciplines. The advancement of synthetic biology technology is contingent upon foundational research, while the commercialization of scientific discoveries necessitates a well-established industrial framework and supportive government policies. In recent years, China has intensified its efforts in fundamental research, industrial development, and policy support for synthetic biology; however, the field remains in its early stages. Its development needs to focus on the following aspects: (a) the existing metabolic pathway prediction and model design of the modification toward high-yielding chassis organisms and developing more universally applicable and effective methodologies for their genetic manipulation; (b) the innovation of equipment and design software at the same time, creating a coordinated intellectual property protection environment; (c) regulatory frameworks need to manage risks and ensure an appropriate balance in the process of industrialization of synthetic biology technology; (d) application standards and norms should be clarified in a timely manner, and regional and global cooperation and standardization are essential to advance the field of synthetic biology and strengthen biosecurity defenses.

## Data Availability

Data sharing not applicable to this article as no datasets were generated or analyzed during the current study.
